# Differential Effects of Classical vs. Sports Massage on Erector Spinae and Upper Trapezius Muscle Stiffness: A Shear-Wave Elastography Study in Young Women

**DOI:** 10.3390/sports12010026

**Published:** 2024-01-09

**Authors:** Amadej Jelen, Erina Javornik, Manca Zupančič, Žiga Kozinc

**Affiliations:** 1 Faculty of Health Sciences, University of Primorska, Polje 42, SI-6310 Izola, Slovenia; 2 Andrej Marušič Institute, University of Primorska, Muzejski trg 2, SI-6000 Koper, Slovenia

**Keywords:** massage, muscle stiffness, elastography, back muscle, relaxation

## Abstract

Classical and sports massages are commonly used interventions, but their comparative effects on muscle stiffness remain unclear. Classical massage is more general and uses light to moderate pressure, and its main purpose is relaxation. Sports massage, on the other hand, is more specialized and targets the unique needs of massaged individuals using moderate to firm pressure. This study aimed to evaluate the impacts of classical and sports massages on the stiffness of the erector spinae (ES) and upper trapezius (UT) muscles. Fifteen recreationally active young women, aged 22.9 ± 1.2 years, underwent a randomized cross-over study (with three conditions). Participants received either a five-minute classical or sports massage or a passive rest as a control on distinct days. Muscle stiffness was assessed using shear-wave elastography. The ES shear modulus displayed a significant time effect (*p* < 0.001; η^2^ = 0.515) without noticeable differences between the conditions, and the time × massage-type interactions approached statistical significance (F = 2.014; *p* = 0.073). There was also a large and statistically significant effect of the time on the UT (F = 11.127; *p* < 0.001; η^2^ = 0.443). We could not prove that classical and sports massages reduced muscle stiffness. The absence of significant differences might be attributed to the specific intervention parameters (massage duration of 5 min) and the small, only young women sample size. Given some tendencies towards significant effects, larger sample sizes are needed to further investigate this research question.

## 1. Introduction

Chronic pain presents a significant personal and economic challenge [1]. In industrialized nations, chronic pain prevalence is alarmingly high. Notably, a systematic review by Fayaz et al. [2] indicated that between 35.0% and 51.3% of people experience chronic pain, with 10.4% to 14.3% struggling with moderate to severe chronic pain. Particularly vulnerable areas are the back and neck [3]. Hogg-Johnson et al. [4] observed that roughly half of the population experiences neck and upper back pain annually. The implications of pain go beyond physical discomfort, influencing socio-economic dynamics and profoundly impacting an individual’s life quality [5]. Given this, the pursuit of cost-effective pain relief interventions is paramount.

Maintaining appropriate muscle stiffness is crucial for effective and functional movement [6]. Ando et al. [7] found a positive correlation between gastrocnemius stiffness and jump performance. Furthermore, muscle stiffness has been linked to muscle strength in elderly women [8]. However, overly rigid muscles can be indicative of numerous neuromuscular and musculoskeletal issues. Examples include spasticity [9], low back pain [10], adolescent idiopathic scoliosis [11], and postural abnormalities [12]. As such, the ideal level of muscle stiffness appears to vary based on the specific muscle, demographic, and situation. Brandt et al. [13] reported a significant connection between neck and upper back pain and trapezius muscle stiffness among office workers. Kocur et al. [14] further determined that female office employees with neck pain exhibited heightened upper trapezius fiber stiffness and pronounced forward head postures. These observations suggest that mitigating trapezius muscle stiffness could potentially alleviate existing pain and reduce the likelihood of future occurrences. Increased muscle stiffness (i.e., erector spinae and multifidus muscles) has also been observed in patients with chronic low back pain [15,16], indicating that they may also benefit from targeted interventions to alleviate muscle stiffness.

Massage therapy, with its rich history and well-established benefits, has been extensively recognized for its capacity to alleviate pain, diminish swelling, counteract stiffness, and enhance muscle mobility [17,18,19]. At its core, massage embodies the rhythmic mechanical manipulation of body tissues, applying a range of pressures and grips [20]. Its impacts go beyond mechanical effects, inducing physiological responses such as reduced spinal excitability, augmented blood circulation, and a rise in the temperature of the treated area [19,21]. It is worth noting that optimal massage pressure depends on individual preferences and the specific condition of the muscle, considering both its biological and pathological state [22]. Among the many massage types, classical or Swedish massage is perhaps the most widely used for relaxation purposes and encompasses techniques such as surface effleurage, deep effleurage, petrissage, shaking, deep rubbing, and tapotement [19,23]. Classical massage has the intention to relax muscles, move body fluids (i.e., lymph and blood), removing wastes from body cells, nourish cells, and diminish any pain [24]. Sports massage shares similarities with classical methods but is often applied more forcefully, which is also a common technique, and it involves deep effleurage, petrissage, tapotement, friction, compressive strokes, broad circular friction, and jostling strokes [25]. No universally accepted definition of sports massage exists, but it is used for either warming up before or during a sports match or for recovery after a match, and it shows the best results when applied a maximum of two hours after exercise [26,27]. The main goal of sports massage is to support fast recovery after exhaustive exercise, and it decreases perceived muscle soreness and fatigue [28,29]. The pace of performing a sports massage technique depends on its purpose, and regeneration massage is performed very slowly while warm-up massage is performed rapidly. Despite the widespread belief in massage’s efficacy in mitigating muscle stiffness, there is a notable gap in the literature probing its impact on muscle stiffness measured objectively, indicating the need for more research in this domain.

In the last two decades, elastography has emerged as an effective technique for assessing the elasticity of soft tissues [30]. In particular, shear-wave elastography offers a non-invasive approach, enabling the real-time imaging of soft tissues and the quantification of their stiffness [31]. Central to this method is the assessment of shear-wave propagation speeds, where stiffer tissues invariably present higher velocities [32]. Based on shear-wave velocity, the shear modulus (kPa) is calculated by assuming a constant tissue density (1000 g/dm^3^ for muscle tissue). Research using shear-wave elastography has yielded encouraging outcomes when examining a spectrum of traumatic and pathological conditions in musculoskeletal soft tissues, including tendons [33], muscles [34], nerves [35], and ligaments [36]. Yet, this technique has been scarcely used for discerning the effects of massage on muscle stiffness.

A study that implemented a seven-minute classical massage protocol observed a decrease in the gastrocnemius shear modulus immediately post-massage [37]. However, the values returned to their initial levels within only 3 min. Furthermore, Pérez-Bellmunt et al. [38] applied a five-minute warm-up sports massage to the gastrocnemius, noting an elongation in muscle contraction time and potentially signifying reduced stiffness. While such results hint at the potential influence of classical and sports massage on muscle stiffness, extrapolating these findings to other muscle groups is questionable. As mentioned earlier, a stiffer gastrocnemius has been correlated with a better athletic performance [7], bringing into question the motive behind massaging this specific muscle. Moreover, it is imperative to evaluate the impact of massage on stiffness across different muscles. This study specifically targeted the back and neck muscles due to documented instances of increased stiffness in these areas among chronic pain sufferers [15,16].

In this study, the primary objective was to investigate the acute effects of two distinct massage techniques, classical and regeneration sports massage, on the stiffness of the upper trapezius and erector spinae muscles, as measured by shear-wave ultrasound elastography on young, recreationally active women. Drawing on prior research, our primary hypothesis was that both massage methods would substantially decrease stiffness (i.e., the shear modulus) in the aforementioned muscles [37,38]. Moreover, we postulated that noticeable changes would be observed immediately after the massage and that the effects would likely disappear within minutes [37]. Although the two massage techniques have not been directly compared before, we also hypothesized that sports massage is more effective than classical massage due to its targeted application and intensity [25,27]. Notably, the existing literature lacks studies comparing the impacts of various massage types on back and neck muscle stiffness as assessed by objective methods such as elastography. This study was the first to compare the effects of different massage techniques on objectively measured muscle stiffness in the back and neck areas. As a secondary question, we investigated a potential correlation between perceived massage-induced pain, relaxation, and alterations in muscle stiffness.

## 2. Materials and Methods

### 2.1. Participants

Based on a previous study [37], a moderate effect (Cohen’s d = 0.66) from massage was expected. We used G*Power 3.1 software (Heinrich Heine University, Düsseldorf, Germany) for calculating the sample size for a within-factors analysis of variance (ANOVA) (effect size (f) = 0.33; α error = 0.05 and power = 0.80). The Cohen’s d value (0.66) was converted to a Cohen’s f value (0.33) to accommodate the input parameters in the software. This computation recommended a sample size greater than 13 participants. Therefore, a convenience sample of 15 healthy and recreationally active female participants (aged 22.9 ± 1.2 years; body height of 164.4 ± 6.4 cm; and body mass of 58.7 ± 6.2 kg). They had no current or previous neck or back injuries, no current musculoskeletal conditions, no pain complaints, no myopathies, and no neurological disorders. The participants were informed about the course and purpose of the research and they also signed a declaration of voluntary consent to participate. They were asked to refrain from intensive resistance training for at least 48 h before each measurement. The research methods and interventions used were non-invasive and approved by the Commission of the University of Primorska for Ethics in Human Subjects Research.

### 2.2. Study Design

Our research employed a randomized cross-over design. The participants made three separate visits to the laboratory, each for the following distinct experimental conditions: classical massage, regeneration sports massage, and a control condition. We employed a quasi-randomized design to determine the sequence of interventions—classical massage, regeneration sports massage, and control—as well as the order in which the muscles were tested. We utilized a Latin square design to systematically vary the order of the massage techniques and the sequence of the muscle testing, thereby mitigating the potential confounding effects of the treatment order. The sequence of these conditions and muscle order-erector spinae (ES) and upper trapezius (UT) was randomized during the first visit using a blind draw from an envelope that contained a limited number of combinations based on the Latin square method. To ensure consistency and mitigate the potential influence of circadian rhythms, each participant underwent all measurements approximately at the same time of day. All assessments were conducted in a consistent environment, specifically, in an air-conditioned room with the temperature maintained between 22 and 23 °C.

First, the participants were asked to lie prone on the massage table and to completely relax for 5 min before the measurements. This was done to reduce the effects of previous activities on muscle stiffness. Following this, we utilized shear-wave elastography to measure the stiffness of both muscle groups. The designated muscle (either ES or UT, depending on the randomization) was then subjected to massage. During the control condition, instead of a massage, the participants remained relaxed in a prone position for 5 min. A study had indicated that merely lying on a massage table could reduce anxiety and cortisol levels [39]. Thus, this control condition was essential to discern if passive rest alone impacted ES and UT stiffness. Subsequent to the massage (or passive rest for the control condition), we remeasured the stiffness of the massaged muscle immediately, followed by additional measurements at 5 and 10 min post-massage. After these measurements were completed for the first muscle, we proceeded with the massage and subsequent measurements for the second muscle. To gain insights into the participants’ subjective experiences, they rated their relaxation levels pre- and post-massage on a scale of 0 (not relaxed at all) to 10 (maximum relaxation). Post-massage, they also evaluated the pain experienced during the massage on a scale ranging from 0 (no pain) to 10 (worst imaginable pain) [40].

### 2.3. Massage Intervention

The massage intervention was performed by a graduate kinesiologist with several years of experience with various massage applications. Both massage techniques were performed on both muscles for 5 min using 5 techniques, each of which was performed for one minute. The massage pressure was defined on a five-point scale, from light lotioning (grade I), heavy lotioning (grade II), medium pressure (grade III), strong pressure (grade IV), and deep pressure (grade V). In a similar manner, we defined the massage tempo as extremely slow paced (grade I), slow paced (grade II), a moderate pace (grade III), fast paced (grade IV), and a rapid pace (grade V). We used this system to specify the amount of massage pressure and the tempo, in this order, for every technique later on.

A classic massage of the ES consisted of surface effleurage (I and IV) with the whole palm perpendicular to the spine from the opposite side, deep effleurage (II and III) with the root of the palm and the fingers in the direction of the feet to the head, rubbing (III and III) with the fingers in the direction of the the head to the feet, petrissage (III and II) with the root of the palm at an angle of 45 degrees to the spine in the direction of the feet to the head, and deep effleurage (II and III) with the whole palm in the direction of the head to the feet. A classic massage of the upper trapezius fibers consisted of kneading (I and IV) the muscle perpendicular to its course with the fingers, surface effleurage (II and III) with the whole palms from the direction of the acromion towards the C7 vertebra, deep effleurage (III and III) with the whole palms from the direction of the acromion towards the C7 vertebra, kneading (III and II) with the fingers in the direction of the acromion towards the C7 vertebra, and rubbing (II and III) with the palms in the direction of the C7 vertebra towards the acromion.

A sports massage of the ES included deep effleurage (III and III) with the little finger side of the palm in the direction of the feet to the head, kneading (IV and II) with the root of the palm in the direction of the spine, deep petrissage (V and I) with the root of the palm and the knuckles of the other hand in the direction of the head to the feet, deep rubbing (IV and I) with the thumbs perpendicular to the spine, and deep rubbing (IV and I) with the root of the palm at an angle of 45 degrees to the spine in the direction of the feet to the head. Sports massage of the upper trapezius fibers consisted of deep effleurage (III and III) with the thumb side of the palm in the direction of the acromion towards the C7 vertebra, kneading during deep effleurage (IV and II) with the fingers in the direction of the acromion towards the C7 vertebra, deep rubbing (V and II) with the knuckles in the direction of the C7 vertebra towards the acromion, kneading (IV and I) the muscle perpendicular to its course with the fingers, and deep rubbing (IV and II) with the little finger side of the palm in the direction of the C7 vertebra towards the acromion.

### 2.4. Muscle Stiffness Assessment

**Figure 1:**
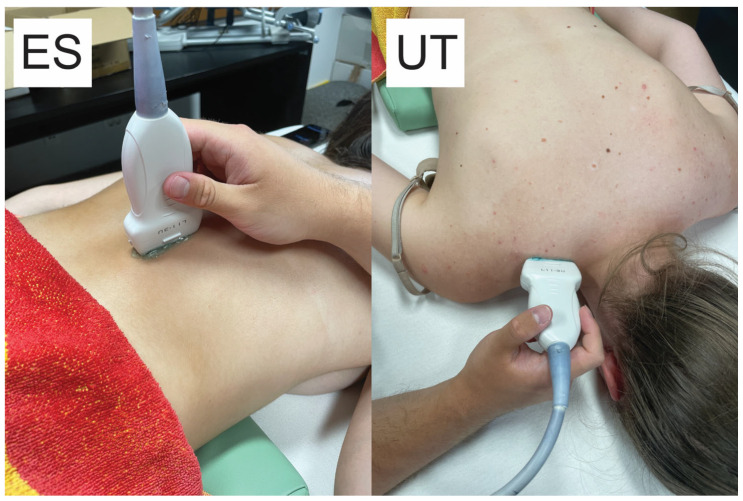
Snapshots of the ultrasound measurements showing the probe positioning on a participant. ES, erector spinae; UT, upper trapezius.

**Figure 2:**
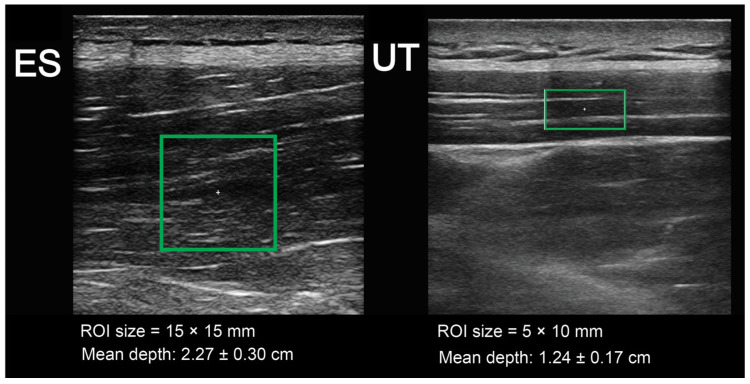
Screenshots of the ultrasound measurements with the regions of interest shown on the muscle tissues. ES, erector spinae; UT, upper trapezius.

Ultrasound assessments were performed by an examiner with ample experience in shear-wave elastography. The examiner was blinded to the randomization of the conditions. Muscle stiffness was measured using a diagnostic ultrasound system (a Resona 7 (Midray, Shenzhen, China)) using the shear-wave elastography method. The system was set to the musculoskeletal mode (MSK) (assuming a muscle tissue density of 1000 kg/m^3^) [41]. We used the medium-sized linear probe model L11-3U (Midray, Shenzen, China) with a water-soluble hypoallergenic ultrasound gel (AquaUltra Basic–Ultragel, Budapest, Hungary). The region of interest area was set to 15 × 15 mm for the ES and 5 × 10 mm for the upper trapezius muscle (Figure 1). The depths of the regions of interest were set individually to avoid including non-muscular tissues, and the data were recorded to be used in subsequent visits. The measurement location was selected for each subject in accordance with the SENIAM recommendations for EMG measurement, and this was the same in all visits. For the ES, the center of the probe was placed in the line with Th12 vertebrae, with a medio-lateral position chosen as the sport with the greatest prominence. For UT, the probe was placed parallel to the arrangement of the muscle fibers, approximately 1 cm lateral to the neck. The pressure applied to the skin with a probe was very light, while the ultrasound gel remained in contact with the skin and the probe. Muscle stiffness was expressed as a shear modulus (in kPa). The data were entered into prepared tables immediately after collection. Two repetitions of the measurements were performed, each consisting of eight scans in rapid succession, with the mean value determined (Figure 2). The mean value of the two repetitions was then taken for further analyses. The software immediately displayed the shear modulus values, which were then transcribed.

### 2.5. Statistical Analysis

The statistical analysis was conducted using SPSS software (IBM, Armonk, NY, USA, version 26.0). The normality of the data distribution was checked with a Shapiro–Wilk test. The descriptive statistics were provided as means and standard deviations. Intra-class correlation coefficients (ICC) with the two-way random single-measure model (i.e., ICC_2,1_) for absolute agreement were used to assess the relative reliability of our outcomes. To test the main hypothesis, a two-way analysis of variance for the repeated measurements was performed, with condition as the first factor (classic, sports, or control condition) and time as the second factor (before the massage, after the massage, 5 min after the massage, and 10 min after the massage). We expressed the effect size with a partial η^2^. If the main effects were statistically significant, a one-way repeated measures analysis with a Bonferroni correction was performed within each condition, and post hoc paired *t*-tests with Bonferroni corrections were performed between the individual time points, with the effect sizes expressed as Cohen’s d values. The relationship between perceived pain during massage and the subsequent reduction in back muscle stiffness was assessed using either Pearson’s correlation coefficients or Spearman’s rank correlation coefficients, depending on whether the data followed a normal distribution. Similarly, the link between the sensation of relaxation post-massage and the alleviation of muscle stiffness was analyzed using the same statistical methods. Statistically significant correlations, differences, and effects were confirmed at a confidence level of *p* < 0.05.

## 3. Results

The within-day reliability (repetition by repetition) was excellent for the UT muscles (ICC = 0.92) and good for the ES muscles (ICC = 0.89). The test–retest reliability (between sessions) was good for both muscles (ICC = 0.86) for ES and 0.84 for UT.

### 3.1. Baseline Muscle Stiffness Measurements

There were no baseline differences in the muscle stiffness between the conditions for the ES muscles (classical massage: 17.63 ± 3.22 kPA; sports massage: 16.9 ± 3.4 kPA; control group: 16.31 ± 1.8 kPa; *p* = 0.467). There were also no differences for the UT muscles (classical massage: 6.92 ± 1.77 kPA; sports massage: 7.04 ± 1.87 kPA; control group: 6.97 ± 1.36 kPA; *p* = 0.979). The average depths of measurement were 2.276 ± 0.046 mm for ES and 1.240 ± 0.025 mm for UT.

### 3.2. Effects of Classical and Sports Massage on Muscle Stiffness

**Figure 3:**
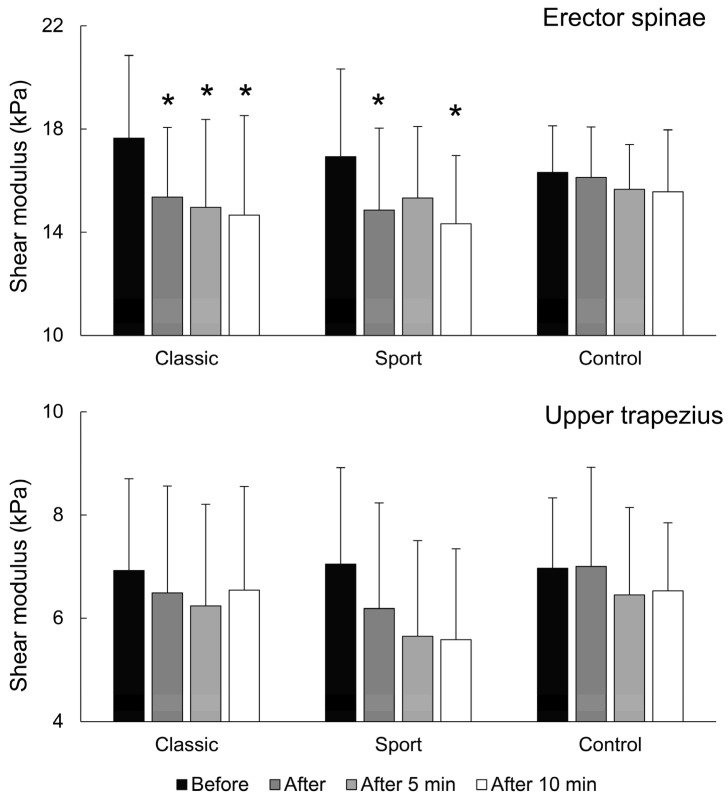
Shear modulus values across the conditions and time points for both muscles. * indicates statistically significant pair-wise difference from baseline within each condition (Bonferroni-corrected post hoc test). We note that a main effect of time was also present for sports massage for the UT muscle (not indicated on the figure).

For ES muscle stiffness, there was a large and statistically significant effect of time (F = 14.891; *p* < 0.001; η^2^ = 0.515), but there were no effects for massage type (F = 0.377; *p* = 0.68), nor were there effects for time × massage-type interaction (F = 2.014; *p* = 0.073). For classical massage, the pair-wise post hoc tests indicated that ES stiffness was reduced immediately after massage (*p* = 0.027; d = 0.76), 5 min after massage (*p* = 0.037; d = 0.81), and 10 min after massage (*p* = 0.023; d = 0.83). For the sports massage condition, ES stiffness was reduced immediately after massage (*p* = 0.016; d = 0.63) and 10 min after massage (*p* = 0.008; d = 0.85), but not 5 min after massage (*p* = 0.252). For the control condition, the pair-wise post hoc test indicated there was no reduction in any time point (immediately, (*p* = 1.000), 5 min (*p* = 0.620), and 10 min afterward (*p* = 0.280) (Figure 3, upper panel).

For the upper trapezius muscle, there was a large and statistically significant effect for time (F = 11.127; *p* < 0.001; η^2^ = 0.443), but there were no main effects for the massage type (F = 1.33; *p* = 0.281) or time × massage-type interaction (F = 1.299; *p* = 0.284). However, the pairwise tests for sports massage approached statistical significance for the differences between the baseline and 5 min (*p* = 0.077; d = 0.75) and 10 min (*p* = 0.069; d = 0.81) after massage (Figure 3, lower panel).

### 3.3. Perception of Relaxation and Association with Changes in Shear Moduli

There was a large and statistically significant effect for time (F = 38.128; *p* < 0.001; η^2^ = 0.731), massage type (F = 4.345; *p* = 0,023; η^2^ = 0.237), and time × massage-type interaction (F = 10.286; *p* = 0.002; η^2^ = 0.424) for feelings of relaxation before and after massaging the ES. The post hoc tests indicated large and statistically significant increases in relaxation after classical massage (F = 46.411; *p* < 0.001; η^2^ = 0.768) and after sports massage (F = 19.034; *p* = 0.001; η^2^ = 0.576) but not after the control condition (F = 3.027; *p* = 0.104). For the UT, there were large and statistically significant effects for time (F = 45.411; *p* < 0.001; η^2^ = 0.764) and time × massage-type interaction (17.074; *p* < 0.001; η^2^ = 0.549), but there were no main effects for massage type (F = 1.222; *p* = 0.308). The post hoc tests showed that there were large and statistically significant increases in feelings of relaxation after classic (F = 46.508; *p* < 0.001; η^2^ = 0.769) and sports massage (F = 22.892; *p* < 0.001; η^2^ = 0.621) but not after the control condition (F = 3.500; *p* = 0.082).

The Spearman’s rank correlation coefficients showed small but significant correlations between changes in the feelings of relaxation in the ES muscles and changes in the shear moduli for before and after massage (r = −0.34; *p* = 0.023), before massage and 5 min after (r = −0.33; *p* = 0.026), and before massage and 10 min after (r = −0.36; *p* = 0.016). However, there was no correlation between the upper trapezius muscle stiffness and the relaxation reports (r = −0.033 to −0.24; *p* = 0.12 to 0.83).

### 3.4. Pain Ratings

The mean pain ratings for the ES massage were 1.87 ± 0.35 for a classical massage (range = 0–5) and 4.53 ± 0.43 (range = 2–8) for sports massage. For the UT muscle, the mean pain scores were 2.20 ± 0.41 (range = 1–5) for classical massage and 5.27 ± 0.53 (range = 2–9) for sports massage. There were no correlations between the pain scores and reductions in the shear moduli for either massage technique (r = −0.032 to 0.032; *p* = 0.87 to 0.99) for both the ES and (r = −0.088 to −0.079; *p* = 0.64 to 0.68) the UT.

## 4. Discussion

This study aimed to investigate the impacts of both classical and sports massage techniques on stiffness of the ES and UT muscles in healthy young women. Our findings could not confirm that classical and sports massage effectively alleviated stiffness in the ES and UT muscles as there were no significant differences (main effects) between the massage and the passive rest. This occurred because there was also a reduction (albeit a non-significant one, according to the post hoc testing) in stiffness after the passive rest. If the sample size was larger, it is likely that significant reductions in muscle stiffness would have been observed in both massage groups for the ES muscle. For the UT, changes significantly greater than those seen for the passive rest condition could also be confirmed for sports massage with a larger sample. Further, a small correlation was observed between the perceived relaxation level and the reduction in stiffness in the ES muscle. However, no correlation was identified between the changes in muscle stiffness and the perceived pain during the massage. In summary, while this study indicated a potential effect of classical massage (ES) and sports massage (ES and UT) on reducing muscle stiffness, further studies with larger sample sizes are needed to confirm if this effect is significantly different from the effect of passive rest.

The primary finding of this study is that brief massage sessions do not significantly reduce stiffness in the back and neck muscles in young, recreationally active women. While earlier studies exploring the effects of massage with elastography yielded varied outcomes, this could be attributed to the examination of different muscles. For instance, Ariji et al. [22] found no effects from a five-minute alternating classical massage on the masseter and temporalis muscles. Conversely, a thirty-minute trigger point massage on both masseters notably reduced muscle stiffness, with a more pronounced effect on individuals who initially had stiffer masseters [17]. Szymczyk et al. [42] observed a marginal decrease in Achilles tendon stiffness after a one-minute percussive massage, but this was not statistically significant (*p* = 0.073). Further, a brief foam rolling session on the pectoralis major muscle did not alter its stiffness [43]. Yet, 5 min of classical massage and 7 min of warm-up sports massage did decrease the stiffness in the medial gastrocnemius [37,38].

Consistent with our results, Eriksson Crommert et al. [37] found no correlation between pain perceived during classical massage and decreased muscle stiffness. However, an intriguing line of inquiry would be to determine if there is an optimal pain threshold for amplifying muscle stiffness reductions, but this subject requires further study. Olchowy et al. [17] reported a median pain perception score of 7.2 out of 10 during trigger point massages, which exceeded all of the average pain ratings from our experiment. They also found a correlation between post-massage relaxation feelings and reduced muscle stiffness—a correlation we observed for ES but not for UT. This suggests that relaxation sensations could serve as a valuable guideline for optimizing reductions in ES stiffness. Contrastingly, as only sports massage was indicative of reductions in UT stiffness, it is plausible that UT is more responsive to greater pressures. Ariji et al. [22] noted that massage pressure did not influence masseter muscle stiffness. Merging the findings of previous studies with ours highlights that responses to massage pressure can be highly muscle-specific.

To date, studies have shown that decreases in muscle stiffness do not last more than a few minutes. Eriksson Crommert et al. [37], for instance, noted that stiffness in the medial gastrocnemius returned to its original state after only 3 min of classical massage, while Szymczyk et al. [42] recorded a similar return for the Achilles tendon 5 min after percussive massage. In contrast, our findings suggested a more prolonged impact, with the lowest muscle stiffness observed 10 min post-massage and 10 min post-passive rest. In examining other modalities, such as acute stretching interventions, results have also been varied. For the hamstrings, Palmer et al. [44] reported a return to baseline stiffness within 5 to 10 min post-stretching. However, Takeuchi et al. [45] found that after intensive stretching, the decrease in hamstring stiffness persisted for at least 20 min. Moreover, when applying dynamic stretching, Iwata et al. [46] documented reductions in hamstring stiffness that lasted up to 90 min. It appears that the duration of reduced stiffness may be influenced by the specific treatment method, its intensity, and the targeted muscle group. Therefore, further exploration is essential to fully understand the extent of massage’s effects on UT and ES stiffness. Our current research suggested that these effects can last for a minimum of 10 min, offering potential relief for individuals suffering from chronic pain in the back and neck regions. Once the ideal massage duration and intensity are determined, more extended interventional studies can be pursued to determine whether massage can also provide long-term relief from back and neck stiffness and the associated pain.

While this study showed that massage does not significantly reduce stiffness compared to passive rest in back and neck muscles, studies with greater sample sizes could easily achieve different results. Also, further research is needed to elucidate the underlying mechanisms. Eriksson Crommert et al. [37] suggested that the decline in muscle stiffness post-massage could be attributed to several factors. These included reduced motor nerve excitability from the relaxation sensation or possibly local reflex inhibition in the massaged area [47,48]. Muscle stiffness is largely underpinned by the number of cross-bridges between actin and myosin filaments [49]. Massage may disrupt these connections. Another avenue of exploration is the role of temperature as massage might alleviate stiffness by warming muscles. Drust et al. [21] found that massage significantly elevated the temperature of the massaged region. With peak temperatures achieved approximately 25 min post-massage [50], this could shed light on why we observed effects up to 10 min post-massage in our study. There is a possibility that the decline in muscle stiffness might extend beyond the ten-minute mark, meriting further study. Another consideration comes from Portillo-Soto et al. [50], who proposed that a rise in skin temperature could indicate augmented blood flow to that region. This increased circulation might, in turn, reduce muscle stiffness [51]. Interestingly, Wiltshire et al. [52] found that massage temporarily hampered blood flow, but it subsequently increased above the baseline. The timeline of these shifts is still uncertain, but the increase in blood flow could be responsible for the sustained reduction in muscle stiffness observed 10 min after massage in our study. Also, certain evidence has suggested that massage could amplify parasympathetic activity, which is evident in heart rate and blood pressure reductions, which might also contribute to declines in muscle stiffness [19]. Lastly, massage increases positive and decreases negative dimensions of a person’s current emotional states [53].

This study presented certain limitations that warrant acknowledgment. Firstly, the researcher responsible for measuring muscle stiffness was not entirely blinded to the experimental condition due to the slight temperature rise and/or reddening of the massaged muscle. While it was challenging to fully differentiate between classical and sports massage, it was evident when a massage had taken place (resulting in inadequate assessor blinding). Moreover, the participants were aware they were being massaged, which introduced potential bias (inadequate participant blinding). Furthermore, our study exclusively focused on the immediate effects of classical and sports massage, prohibiting any inferences regarding long-term impacts. The duration (5 min per muscle) of the massage could also be a limitation of the study, but we wanted to keep the study ecologically valid, since in most cases, in practice, individual muscles are not massaged for more than a few minutes. Lastly, our study cohort consisted solely of young, healthy, recreationally active females. This restricts the generalizability of our findings to broader demographics such as males, other age groups, or, most importantly, those with specific health conditions. In particular, further research is needed to ascertain if massage can reduce muscle stiffness in patients with neck and back pain, how long it lasts, and whether reductions in stiffness are correlated with reductions in pain.

## 5. Conclusions

This study indicated potential benefits of classical and regeneration sports massage on young, recreationally active women; however, we could not confirm that the effects were statistically different from those of passive rest. In the context of the current body of literature, our study adds novel insights for future research, highlighting that both massage techniques might be able to contribute to reductions in stiffness in the ES, with the UT responding favorably only to sports massage. Additionally, a link between post-massage relaxation and diminished stiffness in the ES was indicated, providing a potential therapeutic avenue to explore in future research. The findings further emphasized that there is a possibility that the impacts of massage on muscle stiffness might persist for at least 10 min, suggesting the possibility of practical implications for patients experiencing chronic pain.

## Data Availability

All collected data are included in the manuscript. Raw data are available upon reasonable request to the corresponding author.
